# Fabrication of Polymeric Hydrogels Containing Esomeprazole for Oral Delivery: In Vitro and In Vivo Pharmacokinetic Characterization

**DOI:** 10.3390/polym15071798

**Published:** 2023-04-06

**Authors:** Irshad Ullah, Ayesha Shuja Farooq, Iffat Naz, Waqar Ahmad, Hidayat Ullah, Shama Sehar, Asif Nawaz

**Affiliations:** 1 Department of Pharmacy, University of Swabi, Swabi 94640, Khyber Pakhtunkhwa, Pakistan; 2 Department of Biochemistry, Science Unit, Deanship of Educational Services, Qassim University, Buraidah 51452, Saudi Arabia; 3 Department of Biology, Science Unit, Deanship of Educational Services, Qassim University, Buraydah 51452, Saudi Arabia; 4 Institute of Chemical Sciences, Gomal University, Dera Ismail Khan 29220, Khyber Pakhtunkhwa, Pakistan; 5 Department of Environmental Engineering, College of Engineering, University of Technology, Salmabad 18041, Bahrain; 6 Advanced Drug Delivery Lab, Gomal Centre of Pharmaceutical Sciences, Faculty of Pharmacy, Gomal University, Dera Ismail Khan 29050, Khyber Pakhtunkhwa, Pakistan

**Keywords:** esomeprazole, sodium alginate, hydrogels, drug design, fourier transform infrared spectroscopy

## Abstract

Hydrogel is one of the most interesting and excellent candidates for oral drug delivery. The current study focuses on formulation development of hydrogels for controlled oral delivery of esomeprazole. The hydrogels were prepared by solution casting method by dissolving polymers in Polyvinyl alcohol (PVA) solution. Calcium alginate, Hydroxyl propyl methylcellulose (HPMC), acrylic acid and chondroitin sulfate were used in the preparation of hydrogels. Fourier transform infrared (FTIR) analysis showed no incompatibilities between drug and excipients used in the preparation of formulations. The hydrogels were characterized for size and surface morphology. Drug encapsulation efficiency was measured by Ultraviolet-visible (UV-VIS) spectroscopy. In vitro release studies were carried out using dissolution apparatus. The formulated hydrogels were then compared with the marketed product in vivo using rabbits. The result indicates that prepared hydrogels have a uniform size with a porous surface. The esomeprazole encapsulation efficiency of the prepared hydrogels was found to be 83.1 ± 2.16%. The esomeprazole-loaded hydrogel formulations showed optimum and Pharmacopeial acceptable range swelling behavior. The release of esomeprazole is controlled for 24 h (85.43 ± 0.32% in 24 h). The swelling and release of drug results make the prepared hydrogels a potential candidate for the controlled delivery of esomeprazole. The release of the drug from prepared hydrogel followed the super case transport-2 mechanism. The in vivo studies showed that prepared hydrogel formulations showed controlled and prolonged release of esomeprazole as compared to drug solution and marketed product. The formulations were kept for stability studies; there was no significant change observed in physical parameters, i.e., (appearance, color change and grittiness) at 40 °C ± 2/75% ± RH. There was a negligible difference in the drug content observed after the stability study suggested that all the formulations are stable under the given conditions for 60 days. The current study provides a valuable perspective on the controlled release profile of Hydroxyl propyl methylcellulose (HPMC) and calcium alginate-based esomeprazole hydrogels.

## 1. Introduction

Oral drug delivery, particularly protein-natured active moiety, has gained great interest for safe and controlled administration to patients [1]. Excellent biocompatibility and diversities of synthetic and natural materials options of hydrogel offer outstanding potential as an oral therapeutic drug delivery system [2]. Hydrogel is basically hydrophilic in nature, having 3-D polymeric matrixes type dosage form that aids in retaining a large portion of water and has the propensity to simulate biological tissues when swelling and retain a large quantity of water and at the same time maintain the physicochemical structure of individual polymer chains [3]. Wichterle and Lím reported hydrogel for the first time [4].

The hydrogels possess hydrophilic functional groups such as hydroxyl, carboxyl and amino groups [5]. These groups are found in polymeric chains of hydrogels and are capable of retaining water molecules in the hydrogel formulations [6].

These polymeric materials swell significantly in an aqueous medium and do not dissolve in water at physiological temperatures and pHs [7]. Because of great drug protection, biocompatibility, spatiotemporal control in the release of the drug and physicochemical properties, the materials of this class are employed for the local delivery of the drug [8]. Hydrogels have the ability to encapsulate and deliver drugs of different natures, including small molecules [9], proteins [10] and nucleic acids [11].

The nature of the polymer side groups (ionic or neutral), structural and mechanical properties (phantom or affine networks), techniques of preparations (copolymer or homo), physical structures (crystalline, semi-crystalline and amorphous) hydrogen-bonded, supermolecular structures and hydrocolloidal) and response to environmental stimuli are just a few of the characteristics that can be used to categorize hydrogel systems. Traditionally, hydrogels can be classified into natural hydrogels and synthetic hydrogels [3]. Gelatin, fibrin, chitosan, alginate and hyaluronic acid-based hydrogels are examples of natural hydrogels [12]. Poly (vinyl alcohol) and poly (ethylene glycol) (PEG) are examples of synthetic hydrogels.

The gelatin-based hydrogels are considered to be semi-synthetic hydrogels and are functionalized as synthetic methacryloyl hydrogels and fall under the third category [13]. The delivery of drugs used in the treatment of cancer has been thoroughly studied up to this point using hydrogel systems [14] and various infectious diseases [15], wound healing activities [16], tissue engineering [17] and applications. Hydrogels are advantageous because they are adaptable drug delivery methods. Humans have used devices that can reside in the stomach cavity for diagnostic and therapeutic purposes [18]. HPMC is a hydrophilic polymer; it swells in an acidic medium and releases the drug at a slower/controlled rate [19]. Hydrogels are receiving increasing attention in bio-applications [20]. Among hydrogels, calcium alginate (Ca-alginate) hydrogels are widely used for their biocompatibility, low toxicity, low cost and rapid fabrication by simple mixing [21]. The combination of polymers helped to control the release of the drug from the formulation for longer period of time.

Thermoplastic and thermoset materials have primarily been utilized for their construction [22]. By providing higher compatibilities with the tissues of gastrointestinal (GI) and, possibly, by offering much-increased safety, it expands the range of available materials for systems manufacturing and withstands and extends the possible applications. Because of their softness, this may minimize polymer content and mucosal damage. This might help in the reduction of side effects and maximizes the capacities of dehydration and rehydration in form factors compatible with ingestions and subsequent gastric retentions upon swellings and controlled drug release, and hydrogels are one class of materials that may have enhanced biocompatibility [23].

In a study, a chitosan-based hydrogel was studied for the treatment of peptic ulcers and mucosectomy-induced ulcers [24]. It was concluded that developed hydrogel could be successfully applied for ulcer-healing hemostatic purposes after colon polypectomy or endoscopic mucosal resection (EMR) for accelerating ulcer healing and preventing re-bleeding [25].

In an ethanol-treated rat stomach, about 50% of the ulcer–adhesive keratin hydrogel can reside within 12 h, while only about 18% of it can do so in an untreated rat stomach [26]. Additionally, Keratin hydrogels expedited the repair of the ethanol-induced stomach ulcer by halting the bleeding, shielding the epithelial cells from destruction by gastric acid, reducing inflammation and encouraging re-epithelization [27].

Esomeprazole (EZL) is one of the proton pump inhibitors and authorized by the US Food and Drug Administration (FDA) is an S-isomer of omeprazole. It is a potent acid inhibitor and is inert at a pH of 7. Zollinger elision syndrome, gastroesophageal reflux disease, erosive esophagitis and other acid-related illnesses are all treated with EZL [28]. Esomeprazole has low water solubility and high permeability [29]. It has better pharmacokinetic (PK) profiling than omeprazole in the management of acid-related problems [30]. It has been studied that developed hydrogel preparations could be suitable carriers for the delivery of esomeprazole for the improvement of bioavailability [31].

The current study provides valuable perspective about the controlled release profile of Hydroxy propyl methylcellulose (HPMC) and calcium alginate-based esomeprazole hydrogels. In this study, esomeprazole hydrogels were prepared by the solution casting method. The novelty of the current study was the formulation and characterization of HPMC-alginate-based hydrogels containing esomeprazole for oral delivery. The use of polymers like Hydroxy propyl methylcellulose–alginate has successfully controlled the release of drugs from the formulations. Chondroitin sulfate was used as a polymer in the preparation of esomeprazole-loaded hydrogel. Acrylic acid was used as a monomer because polymer and monomer linked with each other to form a chain, which resulted in the entrapment of the drug. Methylene bisacrylamide was used as a cross-linking agent and helped in the cross-linking of polymer and monomer. However, Hydroxy propyl methylcellulose (HPMC) and calcium alginate was used as polymers for controlled drug release from esomeprazole-loaded hydrogel preparations. The study depicted that esomeprazole-loaded hydrogel formulations can be best-suitable for oral drug delivery. The result of in vitro studies is in good arrangement and confirms the ability of prepared hydrogel formulations to release the drug in a controlled release fashion. This study paves the way to explore the possibility of esomeprazole-loaded hydrogel formulations for oral drug delivery systems. The study also exhibited that the developed hydrogel preparations could be suitable carriers for the delivery of esomeprazole for the improvement of bioavailability and ulcer protection activity.

## 2. Materials and Methods

### 2.1. Materials

Esomeprazole was used as a model drug and was obtained from Wilsons Pharmaceutical Pvt. Ltd. (Sector 1–9, Industrial Area, Islamabad, Pakistan, +92-51-32653015). Chondriodine sulfate (Molecular weight 475.379 g/mol), acrylic acid (Molecular weight 72.06 g/mol) and methylene bisacrylamide (Molecular weight 154.17 g/mol) were purchased from Dow Chemical Company. (693 Washington St #627, Midland, MI 48640, USA). HPMC (Molecular weight 1261.4 g/mol) and sodium alginate (Molecular weight 1170.93 g/mol) were purchased from Sigma-Aldrich, Inc. (St. Louis, MO, USA, +1-314-771-5765), Sigma-Aldrich Chemie Gmbh (Riedstr, Steinheim, Germany, +49-7329-970) and were used as rate controlling agents in the preparation of esomeprazole-loaded hydrogel preparations. Dialysis membrane (Sigma, D-9652; cellulose membrane, avg. flat width 33 mm) purchased from Sigma-Aldrich (3050 Spruce Street, St. Louis, MO 63103, USA) for in vitro drug release study.

### 2.2. Preparation of Hydrogels

Chondroitin sulfate (CS), Alginate (AL) and Acrylic acid [31] and HPMC in various concentrations were mixed with a fixed quantity of polyvinyl alcohol (PVA) for the production of different hydrogel formulations. Solution-casting method was applied, and the temperature was maintained at 50–60 °C. CS and Al were accurately weighed and dissolved in (50 mL) of distilled water and stirred in separate beakers at 50 °C and 50 rpm. PVA was dissolved in 20 mL distilled water and stirred at 90 °C until uniform mixing occurred. Firstly, the PVA solution was poured into the solution of Al. After a few minutes, the mixture was added to the CS solution, followed by the addition of Aa and stirred for 15 min. N′,N′-Methylene bisacrylamide (MBA) was dissolved in distilled water, and ethanol mixture at 50 °C, MBA was then added dropwise into the above-mentioned mixture. The whole mixture was stirred until a translucent solution was formed [32]. All three solutions, after complete solubility, were incorporated and placed for time period of 2 h. In the petri dishes, the blended hydrogels were kept for drying at 40 °C [33]. The composition of prepared hydrogels are shown in Table 1.

### 2.3. Fourier Transform Infrared Spectroscopy

It is an effective method for determining a substance’s chemical structure. It is founded on the idea that chemical bonds, which make up a substance’s fundamental building blocks, can be stimulated and absorb infrared light at frequencies that are characteristic of chemical bonds. The FTIR spectrometer (L1600300, PerkinElmer, 940 Winter Street, Waltham, MA 02451, USA) was used for FTIR analysis. The spectrum of the formulated hydrogels formulations was recorded between 400 cm^−1^ to 4000 cm^−1^ at 32 scans/min. Zinc selenide was used for analyzing spectrum of formulated hydrogel formulations (Mandru, 2019 #30).

### 2.4. Scanning Electron Microscopy (SEM)

SEM became the go-to technique in the morphological evaluation of hydrogels based on its distinct ability to swiftly deliver reliable, detailed information regarding morphology, porous topology, cross-linking status, homogeneity, size, shape and others. Its usefulness is unparalleled, especially when it comes to the porous-directed appearance, integrity, organization, quality and uniformity of such materials. The swelled hydrogel was dehydrated and freeze-dried (usually in liquid nitrogen or by common freezing), and the surface of the sample was covered with a thin conductive layer by sputtering Au, Pd or combinations therefrom. The shape and size of hydrogel were observed by scanning electron microscopy (Jeol 6300) Japan electron optics laboratory company, limited (3-1-2, Musashino, Akishima-Shi, Tokyo 196-8558, Japan) [34].

### 2.5. Swelling Measurements

Accurately weight amount of hydrogels was kept in 30 mL of swelling medium (pH 1.2) at 37 °C in shaking water; after every 15 min, the swelled hydrogels formulations were weighed. The swelling index was determined by the formula as shown in Equation (2). Where Ws and Wd are the weight of the dried and swollen hydrogel, respectively. The study was carried out in triplicate, and results were expressed as mean ± SD to minimize the chances of errors [25].

(1) $$\mathrm{Swelling}\;\%=\frac{\mathrm{Ws}\;–\;\mathrm{Wd}}{\mathrm{Wd}}\times 100$$

where Ws is the weight after swelling, and Wd is the weight in dry state of the hydrogel.

### 2.6. Surface Roughness of Hydrogel

The surface roughness of hydrogel was determined using the processing software ImageJ (NIH, Bethesda, MD, USA). The SEM image of each formulation was selected at appropriate magnification (500×) and processed for the calculation of roughness values quantitatively using the specific plugin. Five readings were taken, and the results were averaged.

### 2.7. Drug Content Analysis

For the determination of the drug content of hydrogel solid particles, 100 mg dried powder of hydrogel was placed in 30 mL phosphate buffer 7.4 at room temperature and stirred for 2 h. The suspension was centrifuged at 5000 rpm for 30 min. The supernatant was analyzed on UV-visible spectrophotometer at 275 nm to obtain free drug, and the sediment was dissolved in 0.1 N HCL and analyzed on UV-visible spectrophotometer (Shimadzu 1801, 1, Nishinokyo Kuwabara-cho, Nakagyo-ku, Kyoto 604-8511, Japan) to obtained the entrapped drug [35].

### 2.8. Encapsulation Efficiency

The hydrogel composites were weighed accurately (0.10 g) using analytical weighing balance. Phosphate buffer (pH 6.8) was prepared in a separate beaker. The weighed amount of hydrogel composites was placed in phosphate buffer solution and stirred continuously for 4 h. After complete mixing, the mixture was placed in sonicator for removal of entrapped air bubbles [36]. The final mixture was centrifuged for 10 min at 10,000 rpm. Following equation was used for the evaluation of % drug encapsulation efficiency (EE) [37].

(2) $$\%\;\mathrm{Drug}\;\mathrm{Entrapment}\;\mathrm{Efficiency}=\frac{Amount\;of\;drug\;in\;Hydrogel}{Theoratical\;drug\;encapsulated}\times 100$$

### 2.9. In Vitro Release Study

The prepared hydrogel formulations were evaluated for in vitro drug release study at pH 1.2 in simulation to stomach pH. The temperature was set at 37 ± 1 °C. In the dialysis membrane (Sigma, D-9652), 10 mL esomeprazole suspension (40 mg hydrogel in 5 mL buffer solutions) was taken. The glass beaker was filled with 20 mL buffer solution, and the membrane was allowed to float freely. In the shaking water bath, the glass beaker was kept at 37 ± 1 °C. The sample from beaker (5 mL) was collected, and fresh buffer solution was placed to maintain sink conditions in the receptor compartment. Spectrophotometrically (UV-VIS Spectrophotometer, Shimadzu 1801, 1, Nishinokyo Kuwabara-cho, Nakagyo-ku, Kyoto 604-8511, Japan) analysis was carried out for collected samples at wavelength of 275 nm [36].

### 2.10. Drug Release Kinetics

Following kinetics models were used for evaluating drug release pattern of esomeprazole from prepared hydrogel formulations [38].

#### 2.10.1. Zero Order Kinetics

This kinetic model is used to evaluate controlled release of drug from formulated dosage form. This model is used to evaluate the constant rate of drug release. This model also helps in evaluating the release of drug that does not disintegrate.

Following equation was used for evaluating zero-order kinetics:

(3) $$\mathrm{wt}=w_{0}\;+\;k_{1}t$$

where “W” represents drug release, “k_1_” represents zero order kinetics and “t” represents relapsed time of drug.

#### 2.10.2. First Order Kinetics

Gibaldi and Feldman (1967) and later Wagner (1969) proposed and used this model for evaluating drug elimination from the biological system. First-order kinetics were used to evaluate absorption, elimination and sink condition.

Following equation was used for evaluating first-order kinetics:

(4) $$\mathrm{logCt}\;=\frac{\mathrm{logCo}\;–\;k_{2}t}{2.303t}$$

where “W” represents release of drug, “k_2_” represents first order kinetics constant and “t” the time required to release the drug.

#### 2.10.3. Hixon–Crowell Model

This method of kinetics was introduced by Hixon–Crowell (1931). This method was used to evaluate changes in the diameter and surface area of the particles.

Following equation was used for evaluating Hixon–Crowell model:

(5) $${\left(100-W\right)}^{1/3}=100^{1/3}-\;k_{3}t$$

where “*W*” represents time required to release the drug from the formulation, “k_3_t” represents surface area, diameter and their representing relationship with Hixon–Crowell kinetic model and “t” represents time of drug release.

#### 2.10.4. Higuchi Model

This kinetic model was first of all introduced by Higuchi and was used for evaluating dissolution rate of the prepared formulations other than ointments.

Following equation was used for evaluating Higuchi model:

(6) $$W_{t}=k_{4}t$$

where “W” represents release of drug from the formulations, “k_4_” represents dissolution rate constants for Higuchi equation and “t” represents release of drug.

#### 2.10.5. Power Law Equation

This equation was first of all proposed and used by Korsmeyer (1983) [39] and Rigter and Peppas (1987) [40]. This is a simple kinetic model and is also known as Korsmeyer–Peppas kinetic model. This model was used to evaluate the relationship between drug release and elapsed time.

Following equation was used for evaluating Power law kinetic model:

(7) $$\frac{\mathrm{Mt}}{\;M\infty }=k_{5}t^{n}$$

where “Mt/M∞” represents release of drug, k_5_ represents Korsmeyer–Peppas kinetic model and *n* represents exponent of diffusion.

### 2.11. In Vivo Studies of Prepared Hydrogel

For in vivo study, approval was taken from Gomal University, Dera Ismail Khan, KP, Pakistan. Male healthy albino rabbits weighing 2–2.5 kg were used for the in vivo studies. The test animals were maintained at room temperature with relative humidity and given standard food. The rabbits were anesthetized with the injection of overdose of ketamine and xylazine [41].

The rabbits were divided into three groups consisting of 5 rabbits per group. Group-A rabbits were given esomeprazole solution, Group-B was given marketed product and Group-C was given optimized hydrogel formulation (F3). Blood samples were collected from the rabbit’s marginal vein at pre-determined time intervals and were centrifuged for collection of plasma. Methanol was added to the plasma and vortexed for 20 min. The vortexed plasma was centrifuged for 3 min at 5000 rpm. The estimation of drug plasma content was carried out on HPLC [42].

### 2.12. Stability Study of Prepared Hydrogel Formulations

The prepared hydrogel formulations were placed for the evaluation of stability studies at accelarated temperature for 60 days at 40 °C/75% RH. This test is of utmost importance for evaluating therapeutic, toxilogical, therapeutic potency of prepared hydrogel formulations. Moreover, this test was also used for evaluating physical, chemical and microbial evaluation of prepared hydrogel formulations [43].

### 2.13. Statistical Analysis

All the experiments were in triplicates, and results averaged (mean ± SD). SPSS version 18 software (IBM, Chicago, IL, USA) was used for statistical analysis. *p* < 0.05 was considered significant. The statistical tool used in the study was one-way ANOVA/post hoc analysis using Tukey’s honestly significant difference test.

## 3. Results and Discussion

### 3.1. Fourier Transform Infrared Spectroscopy

**Figure 1:**
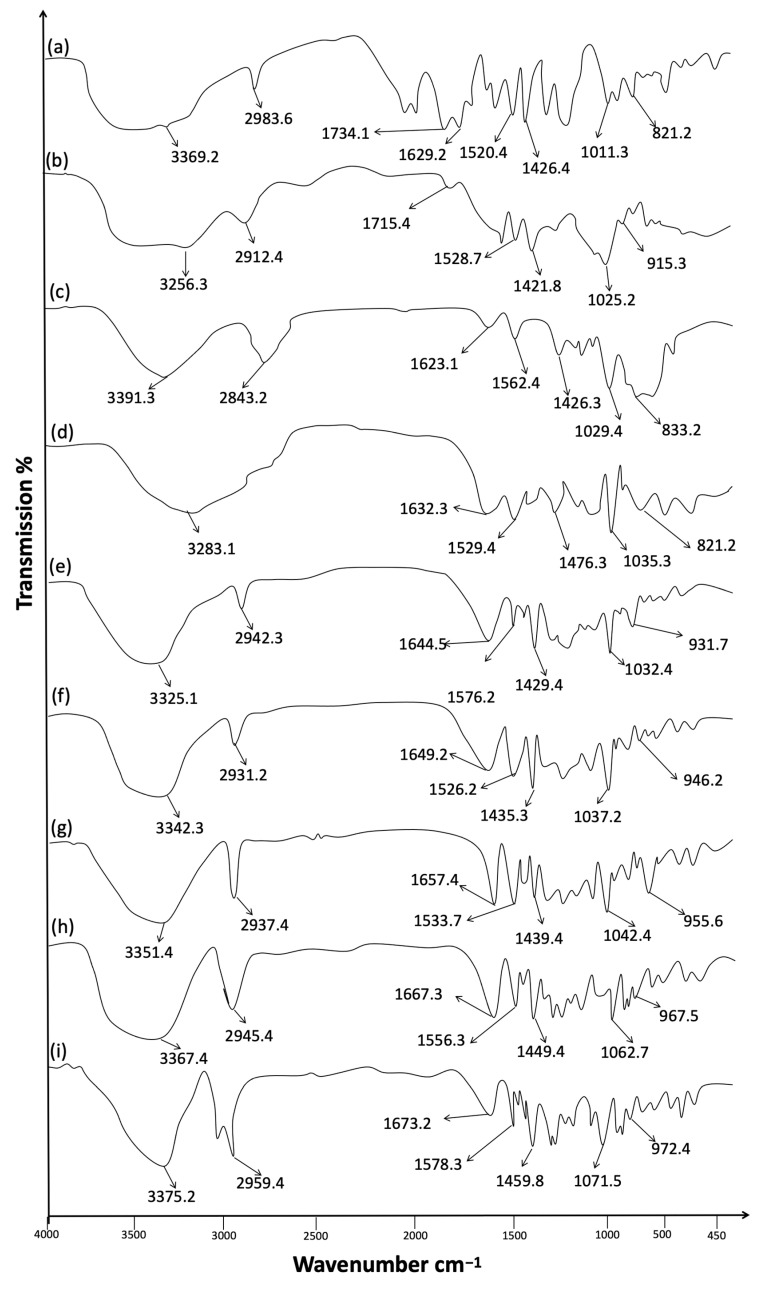
ATR-FTIR spectrum (**a**) Esomeprazole (**b**) Chondotrin sulfate (**c**) HPMC (**d**) Calcium alginate (**e**) F1 (**f**) F2 (**g**) F3 (**h**) F4 (**i**) F5.

FTIR evaluation was used to explore any sort of incompatibilities between drugs and excipients used in the preparation of formulations. The FTIR analysis was carried out for esomeprazole, chondroitin sulfate, HPMC, and calcium alginate individually and also for esomeprazole-loaded hydrogel formulations (F1–F5) (Figure 1) [44]. The spectrum peaks at 3000 cm^−1^–3600 cm^−1^ and 2900 cm^−1^–2950 cm^−1^ were related to OH and CH stretched peaks. The peaks observed in the range 1629 cm^−1^ to 1426 cm^−1^ related to the bending of OH and CH. The bands at 2983 cm^−1^ and 1011 cm^−1^ were linked with the primary alkyl groups (-CH_3_) and ether linkages (-O-CH_2_).

The spectrum showed an additional stretching vibration band at 1734 cm^−1^ and 1629 cm^−1^. The aliphatic carbonyl group confirmed the cross-linkers with polymers. At band 1011 cm^−1^ exhibited sulfonyl group; 812 cm^−1^ for C-N stretched band of secondary amine. The methoxy groups were observed at band 1426 cm^−1^ for methylene C-H bend and a weak band at 2850 cm^−1^ to 2983 cm^−1^. The specific absorption bands of esomeprazole indicated the effective entrapments of esomeprazole in the networks of copolymeric hydrogel formulations. Enhancement of O-H stretching was depicted in a copolymeric hydrogel, which depicted intermolecular hydrogens bond formations within hydrogels formulations. The intermolecular hydrogen bonding depicted extra mechanical strengths to the polymers. The FTIR analysis of prepared formulations exhibited no any sort of incompatibilities between the drug and excipients used in the preparation of esomeprazole-loaded hydrogel formulations (F1–F5).

### 3.2. Swelling Measurements

**Figure 2:**
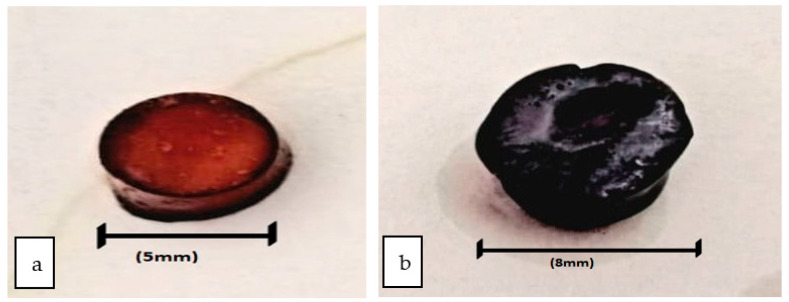
Formulation (F3) Esomeprazole-loaded hydrogel (**a**) Before swelling (**b**) After swelling.

Swelling plays a crucial role in the release of drugs from the polymer matrix. Therefore, the swelling behavior of esomeprazole-containing hydrogel formulations was studied (Figure 2). The swelling behavior of hydrogel formulations revealed optimum swelling behavior. The study depicted that at lower pH, hydrogel formulations are in a unionized state, and the hydrogel bonding formed –COOH groups. This helps the polymers in water uptake and lowers the swelling extent [45].

Among all hydrogel formulations, formulation F3 showed optimum and best suitable swelling behavior, as shown in Figure 2. Before swelling, the maximum size of the hydrogel was 5 mm, as shown in Figure 2a, and an increase in size after swelling was noted up to 8 mm, as shown in Figure 2b. The sustain-release pattern is being followed for the drug release of hydrogels. Hence, the cross-linking of the hard and rigid consistent gels further causes changes in the movement of drug molecules. Their different swelling behavior is responsible for the variances in the cumulative release of the esomeprazole in different pH. Whenever water molecules are penetrated into the network of polymer, then the polymeric strands become uncoiled, which results in the leaching out of the drug from the polymeric network. However, the pH of the release media is responsible for the process of uncoiling the grafted chains. Due to anion–anion repulsion in the basic environment, the relaxation of more chins took place, which further became the cause of the transfer of esomeprazole from the composites of the hydrogel. Due to the formation of hydrogen bonds in the composites of hydrogel, the shrinkage of polymeric segments occurs caused by the acidic environment, which is responsible for the hindrance of the drug release from the composites of the hydrogel [46].

### 3.3. Surface Roughness of Hydrogel

The surface roughness of all the formulations was measured by using the software ImageJ (NIH, Bethesda, MD, USA). The surface roughness of all the formulations ranges from 362.82 nm (0.36282 µm) (Dry form) to 681.22 nm (0.6812 µm) (after hydration). The smooth surface or the minimum rough surface observed indicate that the maximum drug is absorbed in the pores, which reduces the surface’s roughness, while the maximum roughness gives an indication of the less drug attachment to the pore of alginate hydrogels. This surface roughness has a fairly fine finish [47].

### 3.4. Determination of Encapsulation Efficiency and Drug Content

Encapsulation efficiency was carried out for prepared hydrogel formulations, and the study depicted that encapsulation efficiency was higher with increased cross-linking of formulations because the network would strengthen cross-linking and entrap a greater amount of drug within a network of a hydrogel. Higher drug encapsulation efficiency was shown by hydrogel formulation (F3) (83.1 ± 2.16%), which depicted that polymers and cross-linkers preferred drug entrapment in hydrogel formulations (Table 2). Furthermore, the addition of more cross-linking aids in controlling the release of drugs by slowing polymer uncoiling within the medium.

The prepared hydrogels showed drug content values in a uniform manner, and the data obtained lies within official pharmacopeial limits; hence, it is suitable for oral delivery [48]. The prepared hydrogel formulations showed drug content values ranged in between 89.3 ± 1.41% to 92.1 ± 2.31% (Table 2). The maximum amount of drug content was shown by hydrogel formulation (F3) (92.1 ± 2.31%).

### 3.5. SEM Analysis

**Figure 3:**
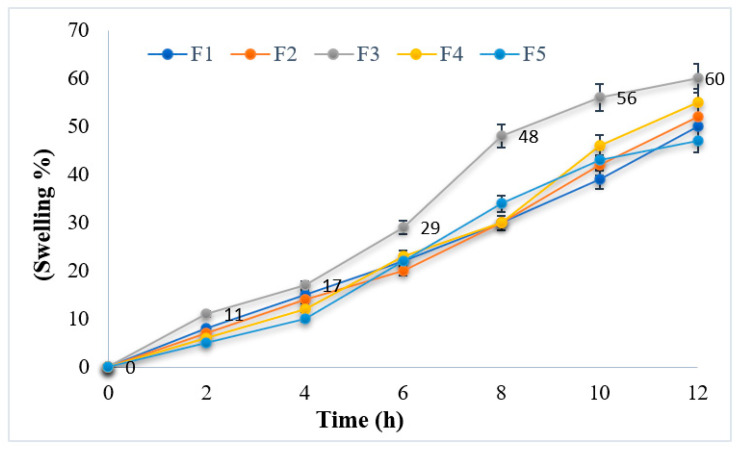
Swelling behavior of esomeprazole-loaded hydrogel formulations (F1–F5).

The surface morphology of the prepared hydrogel formulation was investigated for surface morphology and porosity. The SEM images of esomeprazole containing hydrogel formulations (F1–F5) were revealed to be round-shaped and have smooth surfaces without scratches (Figure 3). The SEM images of prepared hydrogel formulations depicted the existence of highly interconnecting pores and sponges. The pores of prepared hydrogels depicted circular interconnections with spherical shapes (Figure 3). The pores connectivity plays an important role in the fast swelling of hydrogels [49]. The pores connectivity is important in designing drug delivery systems and aids in the permeation of water molecules. This system offers a larger surface area to interact between drugs and molecules of solvents. SEM images of prepared hydrogel formulations depicted drug entrapment in the network of hydrogel formulations (Figure 3).

### 3.6. In Vitro Drug Release Studies

**Figure 4:**
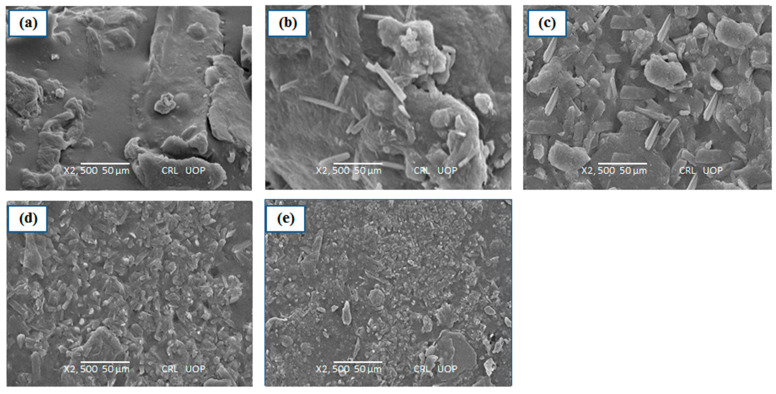
Surface Morphlogy of (**a**) F1, (**b**) F2, (**c**) F3, (**d**) F4, (**e**) F5.

The prepared hydrogel formulations were carried out for in vitro drug release study at pH 1.2 in simulation to stomach pH. The temperature was set at 37 ± 1 °C. In the dialysis membrane (Sigma, D-9652), 10 mL esomeprazole suspension (40 mg hydrogel in 5 mL buffer solutions) was taken. The glass beaker was filled with 20 mL buffer solution, and the membrane was allowed to float freely. In the shaking water bath, the glass beaker was kept at 37 ± 1 °C. The sample from the beaker (5 mL) was collected, and fresh buffer solution was placed in order to maintain the sink condition. The collected samples were analyzed spectrophotometrically using UV visible spectroscopy at 275 nm. The study of in vitro drug release profiles of esomeprazole-loaded hydrogel formulations is shown in Figure 4. The hydrogel formulations have followed the controlled release of the drug. The reason might be due to the presence of polymers and cross-linkers by producing stiffer and rigid gel-like networks and causing the controlled release of drugs from the formulations. The cumulative release of esomeprazole from the hydrogel formulations was carried out for 24 h and showed cumulative release ranged between 59.21 ± 0.21% to 85.43 ± 0.32% at the end of the experiment (24 h).

Among all prepared formulations, formulation (F3) showed the maximum amount of drug release (85.43 ± 0.32%) owing controlled release pattern, while formulation (F1) showed a minimum amount of drug release (59.21 ± 0.21%) shown in Table 3 This is attributed to the presence of varying concentrations of polymers used in hydrogel formulations. The variation in the release of esomeprazole is also attributed to the swelling behaviors of prepared hydrogel formulations. When water is penetrated inside the polymer network, the leaching of the drug from the polymeric network takes place. [44]. In the acidic medium, the hydrogen bond is formed and causes shrinkage of polymer segments in the hydrogel formulations. The study concludes that hydrogel formulation (F3) showed maximum and best-controlled drug release for a time period of 24 h. As in formulation F3, the concentration of calcium alginate is higher, i.e., 1 g, as compared to formulation F5. Both the concentration and the acidic medium (low pH) lead to the hydration of alginate matrices, resulting in the formation of a highly viscous gel layer, which can serve as a drug diffusion barrier. In the presence of certain divalent cations such as calcium (Ca^2+^) or barium (Ba^2+^), alginates also form highly viscous stable gels. The cations act as cross-linkers between carboxyl groups present in the alginate backbone to form a hydrogel network and thereby delay the release of entrapped drug molecules alginate matrix [50].

### 3.7. Kinetic Profiling

Kinetic profiling was carried out using various kinetic models. The data obtained from kinetic profiling was best fitted to the Korsmeyer–Peppas model. The values of drug release exponent (*n*_p_), kinetic constant (*K*) and regression coefficient are presented in Table 4. Dissolution kinetic depicted that value of the diffusion exponent *n*_p_ > 0.89, which depicted that the release of drug from prepared hydrogel formulations was swelling-controlled and related to the relaxation of the polymer during swelling of hydrogel.

### 3.8. In Vivo Studies

**Figure 5:**
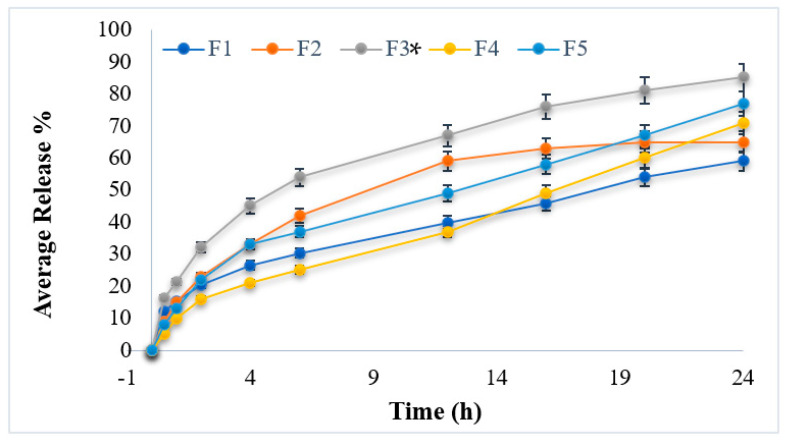
In vitro drug release of esomeprazole-loaded hydrogel formulations (F1–F5). Data are expressed as mean ± SD; *n* = 3. One-way ANOVA followed by post hoc Tukey test (*p* < 0.05), F5 vs. F1. (* *p* < 0.05).

In vivo studies are an important parameter for drug delivery. The optimized formulation (F3) was carried out for in vivo studies (Figure 5). The drug plasma concentration of F3 was compared with the marketed product (positive control) and drug solution (negative control). Different pharmacokinetic parameters such as C_max_, t_max_, AUC, t_1/2_, kel and MRT were investigated and compared.

**Figure 6:**
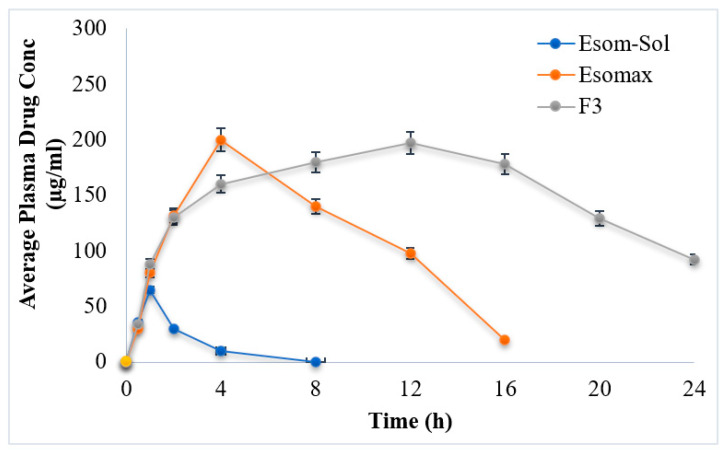
In vivo profile of esomeprazole.

The drug solution (negative control) and marketed formulation (Esomax) showed 97.60 ± 2.31 and 134.72 ± 3.64 µg/mL plasma levels, while the optimized formulation (F3) showed plasma levels of esomeprazole ranging from 209.2 ± 3.57 µg/mL (Figure 6). The biological half-life observed in the case of the marketed formulation (Esomax) was 2 h^−1^, while an increase in the value of biological half-life was observed from formulated hydrogel and ranged between 9.5 h^−1^. This result indicates that the formulated hydrogel will stay longer and will produce controlled effects.

The Esomax and drug solution exhibited the least amount of mean residence time, i.e., 10.73 ± 0.64 h and 3.42 ± 0.39 h, while the hydrogel formulation (F3) exhibited mean residence time ranging between 23.47 ± 0.83 h. The extended mean residence time results in controlled and extended drug activities. The greater AUC values depicted enhanced bioavailability of medication. These results suggest that the prepared hydrogel formulation (F3) delivers the drug at a controlled rate in the presence of polymers such as HPMC and Alginate.

### 3.9. Stability Study of Prepared Hydrogel Formulations

The stability test of prepared hydrogel formulations was placed at an accelerated temperature of 40 °C ± 2/75% ± RH [51]. The result obtained from the stability study revealed that the prepared hydrogel formulations exhibited no significant change (Table 5). There was no change observed in the appearance of color and grittiness. The study also depicted no change in the drug content value, and the result obtained was in accordance with the normal official limits.

## 4. Conclusions

In this study, esomeprazole hydrogels were prepared by the solution casting method. The study depicted that esomeprazole-loaded hydrogel formulations can be best suitable for oral drug delivery. Fourier transform infrared (FTIR) and scanning electron microscopy (SEM) studies confirmed cross-linking of hydrogel formulations. Scanning electron microscopy (SEM) analysis depicted that esomeprazole was properly entrapped in hydrogel formulations. Scanning electron microscopy (SEM) images also depicted that prepared hydrogel formulations have porous structures. The study suggested that the addition of calcium alginate and Hydroxy propyl methylcellulose (HPMC) in equal amounts (F3) was observed as a useful tool for controlling esomeprazole release from prepared hydrogel formulations. The study depicted that esomeprazole in vitro release from prepared hydrogels formulation follows the super case transport-2 mechanism, which refers to the drug release due to polymeric chain relaxation. The result of in vitro studies is in good arrangement and confirms the ability of prepared hydrogel formulations to release the drug in a controlled release fashion, which is also confirmed from the results of in vivo studies. This study paves a way to explore the possibility of esomeprazole-loaded hydrogel formulations for oral drug delivery systems.

## Figures and Tables

**Table 1:** Composition of esomeprazole-loaded hydrogel formulations (F1–F5).

Formulations Code	Esomeprazole (g)	**Chondroitin Sulfate (g)**	Acrylic Acid (g)	Methylene Bisacrylamide (g)	HPMC (g)	Calcium Alginate (g)	PVA (g)	Distilled Water (g)
F1	1	0.2	5	0.05	0.25	1	0.2	17.3
F2	1	0.2	5	0.05	0.5	1.5	0.2	16.55
F3	1	0.2	5	0.05	1	1	0.2	16.35
F4	1	0.2	5	0.05	1.5	0.5	0.2	16.55
F5	1	0.2	5	0.05	2	0.25	0.2	16.3

Abbreviation F1–F5 represents different formulations.

**Table 2:** Encapsulation efficiency and drug content of prepared hydrogels Formulations (F1–F5).

Characteristics	F1	F2	F3	F4	F5
Encapsulation efficacy (%)	81.2 ± 1.27	80.4 ± 1.97	83.1 ± 2.16	79.2 ± 2.41	80.9 ± 1.02
Drug Content (%)	89.3 ± 1.41	91.6 ± 1.32	92.1 ± 2.31	90.8 ± 1.10	90.1 ± 1.21

Data are expressed as Mean ± SD, *n* = 3.

**Table 3:** In vitro release study of esomeprazole-loaded hydrogel formulations.

Characteristics	F1	F2	F3	F4	F5
Average Release (%)	59.21 ± 0.21	65.26 ± 0.31	85.43 ± 0.32	71.63 ± 0.23	77.45 ± 0.23

**Table 4:** Kinetic profiling of esomeprazole-loaded hydrogel formulations.

Formulations Code	Zero Order		First Order		Higuchi		Korsemeyer–Peppas	
	*r* ^2^	*K*_0_	*r* ^2^	*K*_1_	*r* ^2^	*K*_H_	*r* ^2^	*n*_P_
F1	0.935	5.12	0.932	0.023	0.912	4.115	0.934	1.12
F2	0.923	5.32	0.916	0.042	0.932	4.121	0.943	1.34
F3	0.939	5.41	0.927	0.054	0.942	4.154	0.954	1.54
F4	0.919	5.65	0.917	0.017	0.946	4.112	0.923	1.23
F5	0.917	5.15	0.920	0.085	0.965	4.165	0.965	1.56

**Table 5:** Stability of formulations (F1–F5) at accelerated temperature at 40 °C ± 2/75% ± RH.

Formulation Code	Day “0”		Day “30”		Day “60”	
	Color	Drug Content %	Color	Drug Content %	Color	Drug Content %
F1	NC	89.3 ± 1.41	NC	89.1 ± 1.32	NC	88.8 ± 1.41
F2	NC	91.6 ± 1.32	NC	90.8 ± 1.45	NC	91.1 ± 1.32
F3	NC	92.1 ± 2.31	NC	91.7 ± 2.65	NC	91.7 ± 2.31
F4	NC	90.8 ± 1.10	NC	90.1 ± 1.38	NC	89.1 ± 1.10
F5	NC	90.1 ± 1.21	NC	89.9 ± 1.65	NC	89.8 ± 1.21

Data are expressed as mean ± SD (*n* = 3).

## Data Availability

The data presented in this study are available on request from the corresponding author.
